# The Incidence of Distant Metastases in Patients with Pleural Mesothelioma Screened for a Multimodal Approach: How Much Staging Do We Really Need?

**DOI:** 10.3390/cancers16101917

**Published:** 2024-05-17

**Authors:** Arberit Hyseni, Jan Viehof, Jan Hockmann, Martin Metzenmacher, Wilfried Eberhardt, Ken Herrmann, Hubertus Hautzel, Clemens Aigner, Till Plönes

**Affiliations:** 1 Department of Thoracic Surgery and Endoscopy, Ruhrlandklinik, West German Cancer Center, University Hospital, University of Duisburg-Essen, 45239 Essen, Germany; 2 Department of Medical Oncology, West German Cancer Center, University Clinic Essen, University of Duisburg-Essen, 45147 Essen, Germany; 3 Department of Nuclear Medicine, Medical Faculty, University Duisburg-Essen, 45147 Essen, Germany

**Keywords:** mesothelioma, epp, eP/D, epithelioid, MARS trial, imaging, decortication, PM

## Abstract

**Simple Summary:**

This study is an analysis of the metastatic pattern of distant metastases in patients with mesothelioma screened for multimodal treatment and during follow-up. A precise staging of patients undergoing multimodal treatment is mandatory. Distant metastases are more frequent in mesothelioma patients than previously thought. This implies that extensive staging is needed for patients selected for multimodal treatment, including brain imaging and ^18^FDG-PET CT.

**Abstract:**

Pleural mesothelioma (PM) is a very aggressive malignancy with a poor prognosis. Most patients receive systemic treatment only; however, some patients may benefit from multimodality treatment. A precise staging of patients undergoing multimodal treatment is mandatory. We investigated the pattern of metastasis in a cohort of patients screened for multimodal treatment to define the extent of staging examinations. Additionally, we investigated the occurrence of metastasis during follow-up. We investigated a single-center experience of 545 patients newly diagnosed and/or treated with PM between the years 2010 and 2022. Patients who were treated naïvely and had a whole set of imaging of the brain were included and further analyzed. A total of 54% of all patients with cerebral imaging had an available ^18^FDG-PET CT scan. We also recorded metastasis during treatment follow-up. There were 110 patients who had a whole set of imaging (CT = 89% and MRI = 11%) of the brain, and 54% of all patients with cerebral imaging had an available ^18^FDG-PET CT scan. We identified four patients with cerebral metastasis at the time of first diagnosis, which means that 5.4% of the cohort had cerebral metastasis and 13.3% of all patients in the subgroup with complete data of ^18^FDG-PET CT had distant non-cerebral metastasis. During the longitudinal follow-up, we found 11 patients with newly diagnosed metastases after a median time of 1.6 years (range: 2 months to 3.3 years) after first diagnosis without metastases. Distant metastases are more frequent in mesothelioma patients than previously thought. This implies that extensive staging is needed for patients selected for multimodal treatment, including brain imaging and ^18^FDG-PET CT.

## 1. Introduction

Pleural mesothelioma (PM) is a very aggressive malignancy arising mostly years after asbestos exposure from the pleural surface. Due to its rare incidence, there is a lack of large prospective trials showing the most effective treatment [1]. Chemotherapy and, more recently, immunotherapy as palliative treatment approaches extend median overall survival (OS). The recent findings of the MARS2 trial show the importance of the multimodal approach and inclusion of systemic therapy for PM [2]. In selected patients, macroscopic complete resection by either extrapleural pleuropneumonectomy (EPP) or extended pleurectomy/decortication (eP/D) [3] can be performed. These surgical approaches are mostly performed in a multimodal setting in which the surgery is embedded in accompanying (neo-)adjuvant chemotherapy. Even though the multimodal approach is not standardized, currently, chemotherapy with platinum-pemetrexed is most often used [1]. Immunomodulatory drugs embedded in multimodal approaches are currently being evaluated [1]. Even with a selection bias, a subgroup of patients seem to benefit from this approach [4,5]. One of the key points in this concept is adequate patient selection [6]. There are several scoring systems and multiple biomarkers proposed to identify patients with PM who benefit from a multimodal approach. Unfortunately, there are only limited data dealing with pretreatment staging investigations of newly diagnosed mesothelioma patients [7,8,9,10]. Beside local tumor assessment, pre-therapeutic imaging should also detect possible distant metastases with high accuracy and allow the best possible treatment allocation. On the other hand, unnecessary diagnostic procedures may lead to additional radiation exposure, cost, and increased time until treatment initiation. Therefore, pre-therapeutic imaging must be adapted to the risk of finding possible metastases. PM is considered locally highly invasive with a very low tendency for distant metastases. Only a few case reports exist reporting cerebral metastases as a very rare event, mostly at a late stage of disease [11,12,13,14,15]. For this reason, it is questionable whether patients need cerebral imaging in the pre-therapeutic workup. The aim of this study was to investigate the incidence of cerebral metastases in a cohort of patients who were newly diagnosed with PM, treatment-naïve, and screened for multimodal treatment. We also performed a subanalysis of this cohort investigating patients who had ^18^F-fluorodeoxyglucose positron emission tomography/computed tomography (^18^FDG-PET CT) at initial diagnosis for occurrence of non-cerebral distant metastases. We also investigated the longitudinal occurrence of metastases during follow-up.

## 2. Materials and Methods

We investigated all patients diagnosed and/or treated with histologically confirmed PM in our department between the years 2010 and 2022. A histological diagnosis of PM was performed according to IASLC guidelines [16].

Patients who were treated naïvely and had a whole set of imaging (either computed tomography (CT) or magnetic resonance imaging (MRI)) of the brain were included and further analyzed. Beside the incidence of cerebral metastases, we also analyzed if any of the following factors were associated with the occurrence of cerebral metastases: pretreatment serum lactate dehydrogenase (LDH), pretreatment serum C-reactive protein (CRP), and pleural thickness in the initial CT scan (measured at the upper level [extends from the apex of the lung to the inferior margin of the arch of the aorta], middle level [includes pleura between the upper and lower levels], lower level [pleura including and inferior to the first image on which the left atrium is seen], and in the fissure). The analyzed factors proved to be prognostic in earlier studies and are widely available throughout healthcare systems [17,18,19]. The pleural thickness in the CT scan was based on the International Association for the Study of Lung Cancer (IASLC) classification [9,20].

We also performed an analysis of the patient subgroup with initial ^18^FDG-PET CT staging. We recorded the occurrence of distant metastases. We further analyzed the longitudinal occurrence of distant metastases during or after treatment and its impact on median overall survival (OS).

Statistical analysis was conducted by using MedCalc software Version 11.6.1.0 (MedCalc software, Broekstraat 52, 9030 Mariakerke, Belgium). The parameters were analyzed by logistic regression. The difference between different groups or clinical parameters was analyzed by a *T*-test or Mann–Whitney test. A *p* value of <0.05 was considered significant. Survival analysis was performed by a log rank test and Kaplan–Meyer plot. Figures are given as bars or dots.

## 3. Results

**Figure 1:**
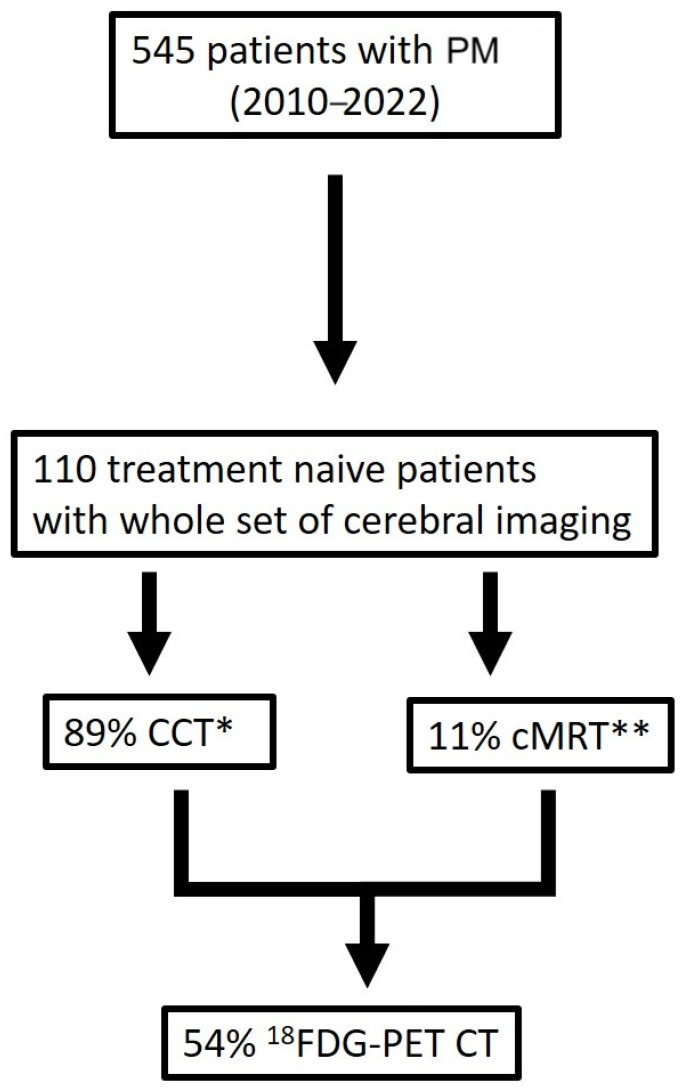
Patients’ selection criteria (* CCT computed tomography scan of the brain, ** cerebral magnetic resonance imaging).

We investigated 545 patients diagnosed and treated with PM in our department between the years 2010 and 2022 (Figure 1). A total of 110 treatment-naïve patients with imaging (CT = 89% and MRI = 11%) of the brain were included and further analyzed. About 54% of all patients with cerebral imaging had an available ^18^FDG-PET CT scan.

The analyzed cohort consisted largely of male patients (87.3%) with the predominantly epithelioid subtype (69.1% epithelioid, 30.9% non-epithelioid [14.5% biphasic, 14.5% sarcomatoid, 1.8% pleomorphic]) and right-sided laterality (57%) (Table 1).

**Figure 2:**
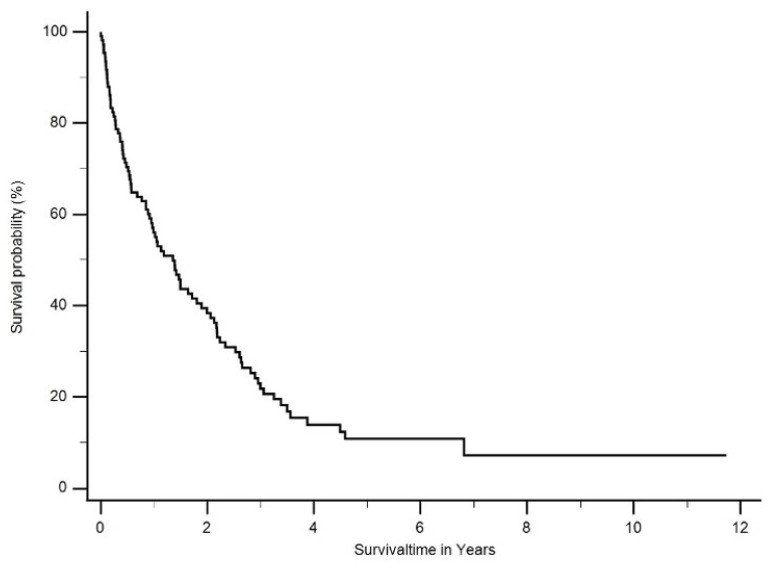
Median overall survival (OS) of all patients was 1.3 years (ranging from 0 months to 11.7 years).

Most patients (69%) were treated by platinum-based chemotherapy. A total of 37.3% (*n* = 41) of patients were selected for multimodal treatment (neoadjuvant cisplatin/pemetrexed followed by EPP or eP/D). Two patients (1.8%) refused further treatment and were excluded from further survival analysis. The median overall survival (OS) of all patients was 15 months (ranging from 0 months to 11.7 years) (Figure 2).

**Figure 3:**
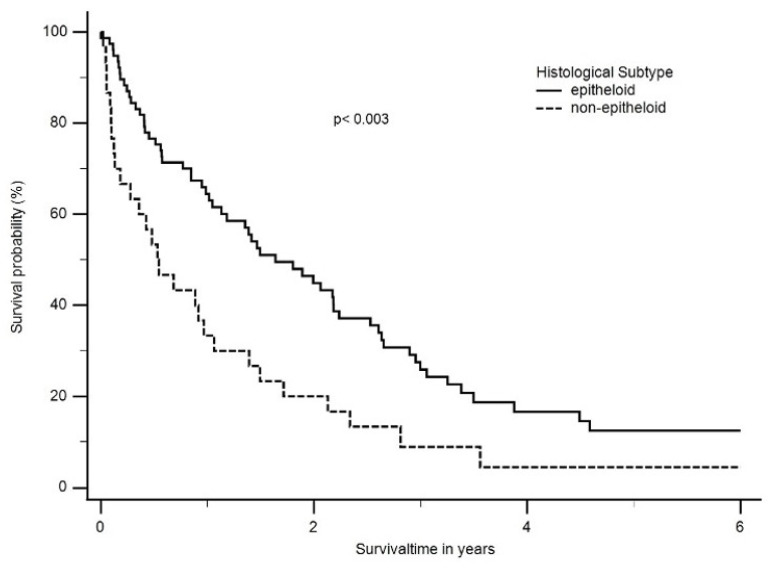
Median overall survival (OS) according to histological subtype. Patients with epithelioid histology had a significant better median OS than patients with a non-epithelioid subtype (1.6 years; ranging from 0 months to 11.7 years vs. 0.5 years; ranging from 4 months to 3.5 years, *p* < 0.003).

For further evaluation, the histological classifications were grouped into epithelioid and non-epithelioid according to the treatment algorithm. Patients with the epithelioid histology had a significantly better median OS than patients with a non-epithelioid subtype (18 months; ranging from 0 months to 140 months vs. 6 months; ranging from 4 months to 42 months, *p* < 0.003) (Figure 3).

**Figure 4:**
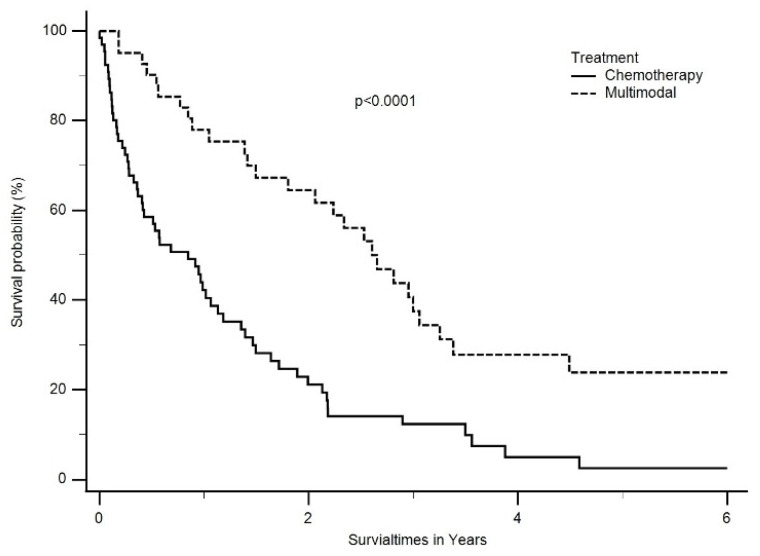
Median OS was also significantly better in patients treated with multimodal treatment than chemotherapy only (2.5 years, ranging from 2 months to 6.8 years vs. 0.8 years, ranging from 0 months to 4.5 years, *p* < 0.0001).

The median OS was also significantly better in patients treated with multimodal treatment compared to chemotherapy alone (30 months, ranging from 2 months to 82 months vs. 9 months, ranging from 0 months to 53 months, *p* < 0.0001) (Figure 4).

The median serum CRP level was 2.1 mg/dL (range: 0.1 to 87.7 mg/dL), the median serum LDH level was 266 U/L, and the median pleural thickness at the upper level was 75 mm (range: 0 to 1250 mm), that at the middle level was 96 mm (range: 0 to 1230 mm), and that at the lower level was 113 mm (range: 0 to 1460 mm).

Cerebral imaging at the time of diagnosis identified four patients with cerebral metastasis, which means that 3.6% of the cohort had cerebral metastasis (Table 2). All patients showed a biphasic subtype. For all four patients, the intended therapeutic treatment changed from multimodal to chemotherapy. In the further analyzed subcohort of patients with complete ^18^FDG-PET CT data at the time of diagnosis, eight patients had one or more distant metastases, mostly in an abdominal organ, the bones, or the lymph nodes outside the thorax, which corresponded to a quota of 7.2%. Most of these patients (*n* = 6) suffered from the epithelioid subtype.

**Figure 5:**
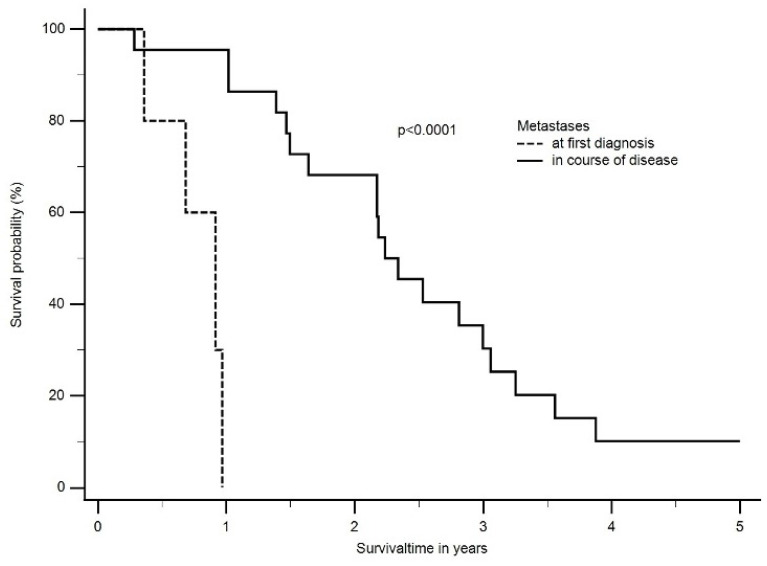
The median OS between patients with diagnosed metastases at time of first diagnosis and patients with metastases diagnosed in the course of disease differed significantly (at time of first diagnosis: 0.9 years; range: 0.2 to 0.9 years vs. metastasis during/after treatment: 2.2 years; range: 0.9 to 3 years, *p* < 0.0001).

In the longitudinal follow-up, 11 patients with newly diagnosed metastases after a median time of 1.6 years (range: 2 months to 3.3 years) were identified. Eight patients were diagnosed with epithelioid and the rest with biphasic mesothelioma. The majority of patients (*n* = 7) were diagnosed in the follow-up period after multimodal treatment, and the most frequent site was the contralateral lung, bones, or intrabdominal organs. The median OS between patients with diagnosed metastases at the time of first diagnosis and patients with metastases diagnosed in the course of the disease differed significantly (at the time of first diagnosis: 0.9 years; range: 0.2 to 0.9 years vs. metastasis during/after treatment: 2.2 years; range: 0.9 to 3 years, *p* < 0.0001) (Figure 5).

In logistic regression, we found no association between the median serum CRP level, the median LDH serum level, the SUV_mean_ of the tumor, or the pleural thickness and occurrence of metastases (neither at the time of diagnosis nor during the course of the disease).

## 4. Discussion

PM is considered locally aggressive with a low tendency for metastatic spread, which increasingly appears more likely in advanced disease [21]. There are only a few published case reports and one recent study dealing with brain metastases in PM; thus, the exact incidence of brain metastases in mesothelioma patients is unknown [22,23]. In the current ESMO guidelines, only CT of the thorax and abdomen is considered the standard staging procedure [1] (Table 3).

Allocation to inadequate treatment may result from excluding imaging from staging. The results show the clinical importance of the detected metastases as a change in treatment regime took place. Especially due to the increase in immunomodulatory therapy options, the staging becomes more relevant. In this cohort, brain metastases at initial staging were only observed in non-epithelioid tumors, whereas distant metastases were observed in both epithelioid and non-epithelioid types. In addition, the current literature does not just represent one histological type [23]. The incidence of distant metastases, however, is of outstanding importance for the selection of staging examinations, which must adapt to the probability of finding a potential distant metastasis. In a register study with 12,516 patients of the Surveillance, Epidemiology, and End Results Database (SEER), He J. et al. analyzed 2138 patients with PM [24]. In this cohort, only 291 patients were reported with distant metastases, but the location of the metastases was not further described. The reported outcome of these patients was associated with a poor prognosis and a negative prognostic factor. Several necropsy studies from Germany, France, and England reported a high prevalence of metastases in all organs [25,26,27,28,29,30,31]. Even if these were often small nodules in postmortem findings, the incidence of metastases seems to be underestimated and more clinically relevant than previously known. This is in line with our findings which indicate that 5.6 percent of all staged patients showed cerebral metastases and 13.3 percent of all patients had blood-borne metastases elsewhere in the body. Also, a recent study indicates an incidence of brain metastases of 4.7 percent. Therefore, other authors also recommend imaging and staging for PM patients. The incidence is therefore approximately 100% higher than previously estimated [22]. Even after treatment, the occurrence of metastases is a clinically relevant problem. In the longitudinal follow-up, we were able to identify metastases in the course of disease in ten percent of all patients. This implies that even after treatment (and especially multimodal treatment), metastases may occur at any time and an appropriate follow-up is mandatory. This is particularly important as local treatment can provide a significant benefit, with an improvement in median overall survival [32]. Therefore, in recent times, PET/CT and cerebral imaging have been performed for all patients considered for multimodal approaches and repeated in the course of therapy. This study is limited due to its retrospective design and the fact that it is a single-center study with a long observation period. Thus, staging algorithms have shifted, and in the recent cohort, PET/CT and cerebral imaging were more frequently performed.

## 5. Conclusions

In summary, we were able to show that patient selection for multimodal treatment should include imaging of the brain and FDG-PET CT. The incidence of metastases is higher than previously assumed. We also suggest periodical follow-up for patients with MPM.

## Figures and Tables

**Table 1:** Clinical characteristics of patients.

Characteristic (*n* = 110)	
Median age (years), mean ± SD	67 (range: 49 to 79)
Sex (male), *n* (%)	96 (87.3%)
Histological subtype, *n* (%)	Epithelioid 76 (69%) Biphasic 16 (14.5%) Sarcomatoid 16 (14.5%) Other 2 (2%)
Treatment, *n* (%)	CTX * 67 (60.9%) Multimodal ** 41 (37.3%) BSC 2 (1.8%) ***
Median CRP ^+^	2.1 (range: 0.1 to 87.7)
Median LDH ^++^	226 (range: 89 to 589)
Side of primary tumor (right), *n* (%)	63 (57.3)
Cerebral metastases at time of diagnosis *n* (%)	4 (5.4%)
Non-cerebral metastases at time of diagnosis *n* (%) ^+++^	8 (13.3%)

* CTX = chemotherapy; ** multimodal treatment consisting of neoadjuvant chemotherapy (four cycles of cisplatin and pemetrexed) and extended pleurectomy/decortication; *** patients refused further treatment and were excluded from follow-up analysis; ^+^ C-reactive protein; ^++^ LDH = lactate dehydrogenase U/L; ^+++^ in a subcohort of 54% with ^18^FDG PET CT.

**Table 2:** Clinical characteristics of patients with metastasis.

Characteristic	
*Cerebral metastasis at initial staging (n = 4)*	
Histological subtype, *n* (%)	Biphasic 4 (100%)
*Distant non-cerebral metastasis at initial staging (n = 8)*	
Histological subtype, *n* (%)	Epithelioid 6 (75%) Non-epithelioid 2 (25%)
Localization of metastasis	Bones *n* = 2 Liver *n* = 3 Cervical lymph node *n* = 1 Colon *n* = 1
*Metastasis during follow-up (n = 11)*	
Median time of diagnosis of metastasis after first diagnosis of mesothelioma	1.6 years (range: 2 months to 3.3 Years)
Histological subtype, *n* (%)	Epithelioid 8 (73%) Non-epithelioid 3 (27%)
Localization of metastasis	Subcutis *n* = 2 Cerebral *n* = 3 Abdomen *n* = 1 Bones *n* = 1 Contralateral lung *n* = 4

**Table 3:** Staging procedures in the screened mesothelioma cohort.

Characteristic (*n* = 110)	
*CCT* *	*n* = 98
cMRI **	*n* = 12
^18^FDG-PET CT *** and CCT/MRI	*n* = 59

* computed tomography scan of the brain, ** cranial magnetic resonance imaging, *** ^18^F-fluorodeoxyglucose positron emission tomography/computed tomography.

## Data Availability

Data supporting the finding of this study are available from the authors on reasonable request.
